# Neonatal leucocyte cell population data: reference intervals and relevance for detecting sepsis and necrotizing enterocolitis

**DOI:** 10.1038/s41390-025-04159-x

**Published:** 2025-06-06

**Authors:** Flavia Ferraro, Laura Fillistorf, Varvara Dimopoulou, Lorenzo Alberio, Christine Coutaz, Sylvain Meylan, Raphael Matusiak, Jeremie Despraz, Eric Giannoni

**Affiliations:** 1 Clinic of Neonatology, Department Mother-Woman-Child, Lausanne University Hospital and University of Lausanne, Lausanne, Switzerland; 2 Service of Hematology and Central Hematology Laboratory, Lausanne University Hospital and University of Lausanne, Lausanne, Switzerland; 3 Infectious Diseases Service, Department of Medicine, Lausanne University Hospital and University of Lausanne, Lausanne, Switzerland; 4 Clinical Data Science, Biomedical Data Science Center, Lausanne University Hospital and University of Lausanne, Lausanne, Switzerland; 5 Swiss Data Science Center, Swiss Institute of Technology, Lausanne, Switzerland

## Abstract

**Background:**

Timely diagnosis of neonatal sepsis is crucial but remains challenging. Cell Population Data (CPD) provide high-resolution phenotyping of leukocytes, offering potential for sepsis detection. We aimed to establish neonatal CPD reference intervals and explore their capacity to detect sepsis and necrotizing enterocolitis (NEC).

**Methods:**

CPD from neutrophils, monocytes, and lymphocytes were analyzed in hospitalized newborns. Reference intervals (5–95th percentiles), at birth and during the first 28 days, were derived from newborns without conditions potentially impacting CPD (reference group). The performance of CPD in detecting blood culture-proven sepsis/NEC was evaluated against complete blood count (CBC) and C-reactive protein (CRP).

**Results:**

Reference intervals from 905 neonates showed that mean CPD values followed distinct trajectories for each parameter, while distribution width generally decreased with increasing gestational and postnatal age. CPD in 39 sepsis/NEC cases, obtained on the day of clinical suspicion or from the closest CBC, differed from those in the reference group, particularly neutrophil fluorescence intensity (NE-SFL) (56.2 vs. 41.1 arbitrary units, *P* < 0.001). NE-SFL had superior accuracy compared to other CPD, CBC, and CRP, with 90% sensitivity and 76% specificity.

**Conclusions:**

This study establishes neonatal CPD reference intervals and identifies NE-SFL as a potential sepsis biomarker.

**Impact:**

- This study establishes reference intervals for neonatal leukocyte Cell Population Data (CPD), providing a valuable resource for interpreting these preclinical parameters in newborns.
- Our findings highlight the impact of gestational and postnatal age on neutrophil, monocyte, and lymphocyte morphology, contributing to a better understanding of neonatal immune development.
- In an exploratory analysis, CPD parameters, particularly NE-SFL, had superior diagnostic accuracy for sepsis and necrotizing enterocolitis compared to traditional biomarkers.
- As CPD are automatically generated with CBC, they offer a cost-effective, real-time, and objective tool with potential for improving neonatal sepsis detection.

## Introduction

Sepsis is a leading cause of death and disability in newborns, with preterm infants being the most vulnerable population.^1^ The rapid progression of neonatal sepsis necessitates early detection to initiate timely empirical antibiotic treatment and optimize outcomes.^2,3^ However, current diagnostic tools have limited accuracy for early identification and differentiation of sepsis from non-infectious conditions.^4^ As a result, some newborns experience delays in receiving antibiotics, while others undergo unnecessary treatment, increasing the risk of adverse outcomes and promoting the emergence and spread of antimicrobial resistance.^5,6^ This highlights the need for novel biomarkers that can assist clinicians in the early and accurate detection of sepsis.

State-of-the-art hematologic analyzers use a combination of impedance and fluorescence flow cytometry to deliver a high-resolution analysis of leukocyte cell volume, complexity, and fluorescence intensity.^7–10^ This detailed information, known as Cell Population Data (CPD), is used in clinical practice to determine the complete blood count (CBC) and leukocyte differential.^10^ Given the critical role of leukocytes in host response to infection, CPD may offer valuable information that could potentially contribute to early detection of neonatal sepsis.^11^ In addition, studying CPD could provide new insights on the developmental biology of blood leucocytes. However, the lack of neonatal reference intervals hinders CPD interpretation. Few studies have suggested the potential of specific neutrophil characteristics as biomarkers for neonatal sepsis.^12–19^ However, no comprehensive evaluation of leukocyte CPD has been undertaken to date. Moreover, previous studies have not leveraged fluorescence flow cytometry technology, underscoring the need for further research in this area.

To address these knowledge gaps, we established reference intervals for leukocyte CPD in a large cohort of newborns. In addition, we explored the potential of CPD as early biomarkers of sepsis and necrotizing enterocolitis (NEC).

## Methods

This single-center, retrospective observational study was conducted in the neonatal unit of the University Hospital of Lausanne from January 2021 to June 2023. It was approved by the Cantonal Ethics Committee of Vaud (Lausanne, Switzerland, ID 2022-00528). The research was conducted in accordance with the relevant guidelines and regulations.

All patients hospitalized in the neonatal unit who had at least one CBC between birth and a corrected age (defined as the sum of gestational and postnatal age) of 44 weeks were eligible, regardless of the clinical indication for the CBC. We excluded newborns whose parents or legal guardians refused general consent for research and those with missing CPD parameters. Neonatal sepsis was diagnosed based on blood culture-proven infection.^20,21^ Contaminated blood cultures were defined by the growth of bacteria typically considered as contaminants (e.g., Micrococcus species or diphtheroids), growth of coagulase-negative staphylococci (CoNS) in patients without a central or peripheral catheter (at the time of blood culture and within a 24 h period prior to blood culture collection), or cultures deemed contaminated by clinicians, leading to discontinuation of antimicrobial therapy within less than 5 days.^21^ NEC was defined as Bell stage 2 or higher.^21,22^ We identified a reference group of newborns without any condition potentially impacting neutrophils, monocytes and/or lymphocytes. The following conditions were considered as potentially impacting CPD: neutropenia (defined as a neutrophil count <1.5 G/L at any time from birth to discharge, regardless of etiology), trisomy 13, 18, and 21, birth asphyxia (initial pH <7.0, 5-min Apgar score <5), hypoxic-ischemic encephalopathy, grade 3 or 4 intraventricular hemorrhage, cystic periventricular leukomalacia, stage 3 or 4 retinopathy of prematurity, and any suspected or proven systemic or focal bacterial, fungal or viral infection leading to antimicrobial treatment for 5 days or more.^23–26^ Data were extracted from the hospital’s clinical information systems, electronic health records, and laboratory information system. CPD parameters were acquired using the Sysmex XN-9000® hematology analyzer which uses a combination of impedance and fluorescence flow cytometry to provide leukocyte differential counts derived from CPD. For neutrophils, monocytes, and lymphocytes, we collected data on side scatter (X-axis), indicating cellular complexity, fluorescence intensity (Y-axis), reflecting nucleic acid content, and forward scatter (Z-axis), denoting cell volume. Additionally, we collected data on the distribution width of each parameter, representing signal heterogeneity. This resulted in a total of 18 CPD variables (Supplementary Table S1).

CPD of neutrophils, monocytes, and lymphocytes obtained from the reference group were analyzed to determine reference intervals, defined as the interval between the 5th and 95th percentiles of observed values, in line with prior studies.^23^ We presented CPD obtained on the day of birth according to gestational age. To provide data on the neonatal period, we presented CPD from the first 28 postnatal days stratified into three gestational age groups: <28 weeks, 28–36 weeks, and >36 weeks.^23,26^ To get insights into the potential of CPD as novel biomarkers, we conducted exploratory analyses of CPD parameters at the onset of sepsis/NEC. We analyzed CPD obtained at the time of blood culture and/or abdominal X ray for NEC. For patients who did not have CPD collected at this time, we analyzed the closest CBC (Supplementary Table S2). We compared CPD from sepsis/NEC cases to neonates in the reference group and applied the same analytical approach to CBC and CRP. We defined leukopenia as a leucocyte count <5 G/L, neutropenia as an absolute neutrophil count <1.5 G/L, and an elevated immature-to-total (I/T) ratio as a ratio >0.2.^27–29^

Patient characteristics were summarized by descriptive statistics. Continuous variables were presented as medians and interquartile ranges (IQR), while categorical variables were expressed as absolute numbers and percentages. Differences between the sepsis/NEC group and the reference group were assessed using Wilcoxon–Mann–Whitney tests for continuous variables and Pearson chi-square tests for categorical variables. To construct reference intervals, data were grouped by gestational age at birth. The median, 5th and 95th percentiles were calculated, and data were smoothed using the Locally Estimated Scatterplot Smoothing (LoESS) technique to provide a continuous and regular representation of trends. A mixed-effects model and Estimated Marginal Means (EMMs) post-hoc analysis was employed to quantify changes in CPD parameters over time and among different gestational age groups. The diagnostic accuracy of each variable was assessed using Receiver Operating Characteristic (ROC) curves and Precision-Recall Curve (PRC) analyses, the latter being better suited in contexts with imbalanced groups.^30^ Area under the ROC curve (AUROC), sensitivity, specificity, positive predictive value (PPV), negative predictive value (NPV), and average precision (AP) were evaluated. A multivariate logistic regression model was developed to predict sepsis/NEC using CRP and the most accurate CPD parameters. The variance inflation factor (VIF) was calculated to assess multicollinearity, and a 10-fold cross-validation ensured robustness. Predicted probabilities were used to generate ROC and PRC curves, with performance metrics quantifying diagnostic effectiveness.

The same statistical analyses (descriptive statistics, non-parametric tests, diagnostic accuracy metrics, and logistic regression) were applied to a population without exclusion of neonates with comorbidities potentially affecting CPD to account for the potential impact of these conditions on the diagnostic performance of CPD parameters. Statistical analyses were carried out using R version 4.3.0. During the preparation of this work the authors used ChatGPT in order to improve readability and language. After using this tool/service, the authors reviewed and edited the content as needed and take full responsibility for the content of the publication.

## Results

### Participants

Among 1693 eligible patients, 512 (30%) were excluded due to missing CPD parameters, and 90 (5%) due to refusal of general consent for research (Supplementary Fig. S1). A reference group of 905 newborns was obtained after exclusion of 135 patients with comorbidities potentially impacting neutrophils, monocytes and/or lymphocytes. We identified 39 newborns with sepsis/NEC. Main demographic, clinical and microbiological characteristics of the study population are reported in Table 1, while those of the population not excluding neonates with comorbidities potentially affecting CPD are provided in Supplementary Table S3.

**Table 1:** Demographics, clinical and microbiological characteristics of patients.

	Reference group *n* = 905	Sepsis/NEC group *n* = 39	*P* value
Postnatal age at sepsis or NEC onset, days	–	9 (6–20)	–
Birth weight, kg	2.8 (2.0–3.4)	0.9 (0.7–2.0)	<0.001
Gestational age, weeks	37 (34–40)	28 (26–34)	<0.001
Female sex	380 (42)	23 (59)	0.05
Pathogens in blood culture proven sepsis	–	34 (87)	–
*Escherichia coli*	–	8 (24)	–
*Coagulase-negative staphylococci*	–	6 (18)	–
*Staphylococcus aureus*	–	4 (12)	–
*Streptococcus agalactiae*	–	3 (9)	–
*Klebsiella pneumoniae*	–	3 (9)	–
*Klebsiella oxytoca*	–	2 (6)	–
*Candida albicans*	–	2 (6)	–
Others pathogens^a^	–	6 (17)	–
Early-onset sepsis	–	8 (24)	–
Late-onset sepsis	–	26 (76)	–
Necrotizing enterocolitis	–	10 (26)	–

Continuous variables are reported as median (interquartile range). Categorical variables are reported as number (percent).

Early-onset sepsis: onset before 72 h of life; late-onset sepsis: onset after 72 h of life.

^a^Achromobacter ssp, Bifidobacterium breve, Clostridium neonatale, Enterobacter cloacae, Enterococcus faecalis, Lacticaseibacillus rhamnosus.

### Reference intervals for CPD parameters

**Fig. 1:**
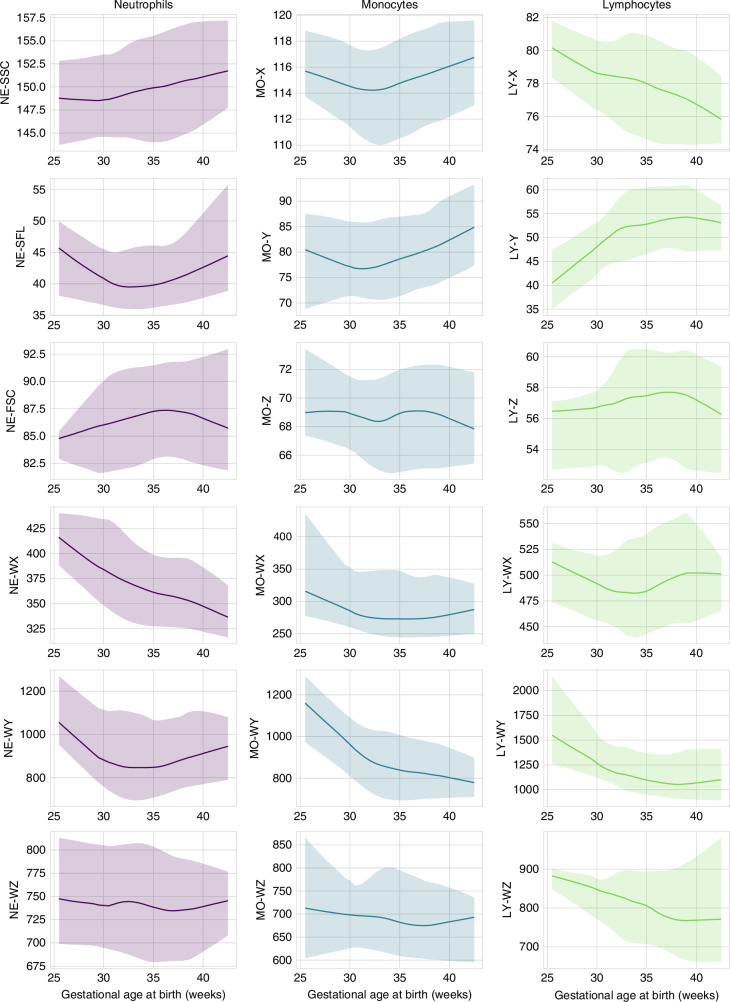
Reference intervals for cell population data on the day of birth. The lines represent the smoothed median values; the shaded areas represent the 5th to 95th percentile range. For lymphocyte CPD, data on 433/905 (48%) patients is provided, as data was not stored in 472 patients.

**Fig. 2:**
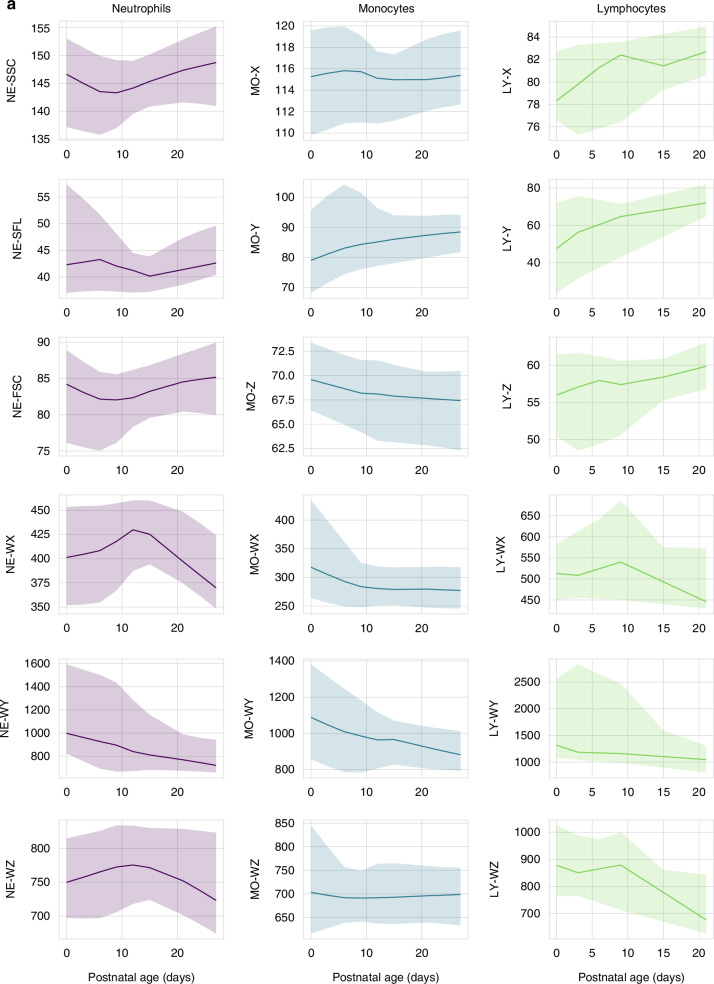

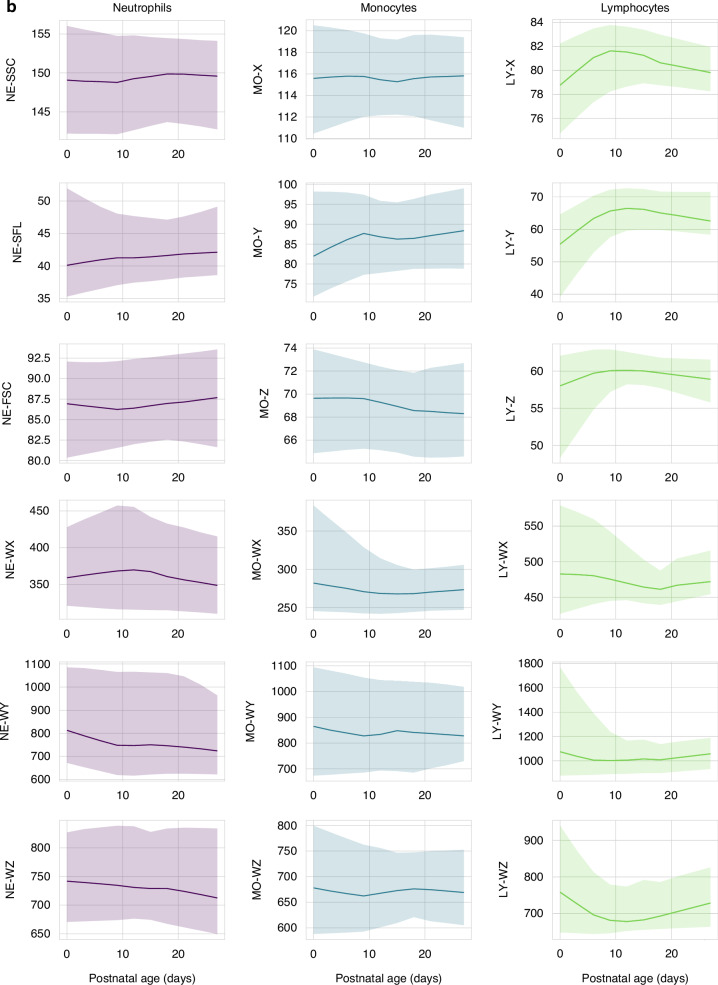

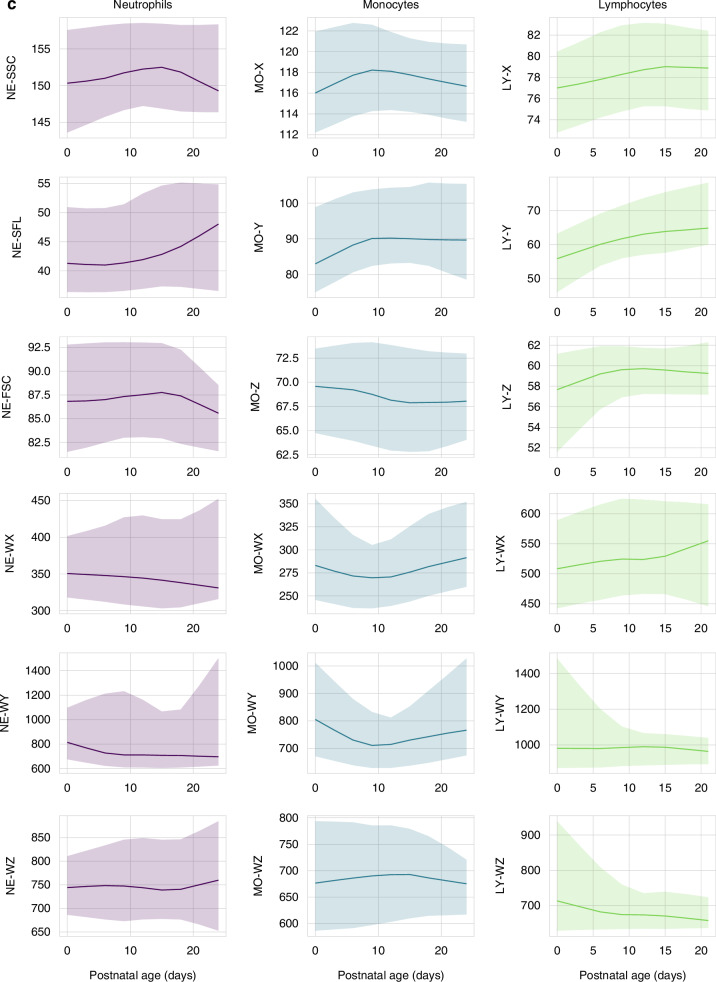
Reference intervals for cell population data during the first 28 days of life. The lines represent the smoothed median values; the shaded areas represent the 5–95th percentile range. **a** Reference intervals for CPD parameters in newborns <28 weeks gestation. **b** Reference intervals for CPD parameters in newborns 28–36 weeks gestation. **c** Reference intervals for CPD parameters in newborns >36 weeks gestation. For lymphocyte CPD, data on 433/905 (48%) patients is provided, as data was not stored in 472 patients.

Reference intervals for CPD parameters were established in the reference group based on gestational and postnatal age. We computed reference intervals for CPD parameters on the day of birth according to gestational age (Fig. 1), and reference intervals over the first 28 postnatal days in three gestational age groups (Fig. 2). CPD parameters varied with gestational age at birth, showing distinct patterns between neutrophils, monocytes, and lymphocytes (Supplementary Table S4). Across neutrophil, monocyte and lymphocyte CPD, we observed a general pattern of reduction in the distribution width of most CPD parameters with increasing gestational age. When analyzing the effect of postnatal age on CPD parameters across the three gestational age groups, we found substantial differences not only in initial values but also in the rate and direction of changes over time (Supplementary Table S5). This analysis also indicated a general pattern of reduction in the distribution width of most analyzed CPD parameters, both with increasing gestational and postnatal age.

### CPD accuracy for the detection of sepsis and NEC

In the sepsis/NEC group, 29 (74%) newborns had culture-proven sepsis, 5 (13%) had NEC, and 5 (13%) had both conditions. Neonates with sepsis/NEC had a lower gestational age and birth weight compared to neonates in the reference group (Table 1, Supplementary Table S3).

**Fig. 3:**
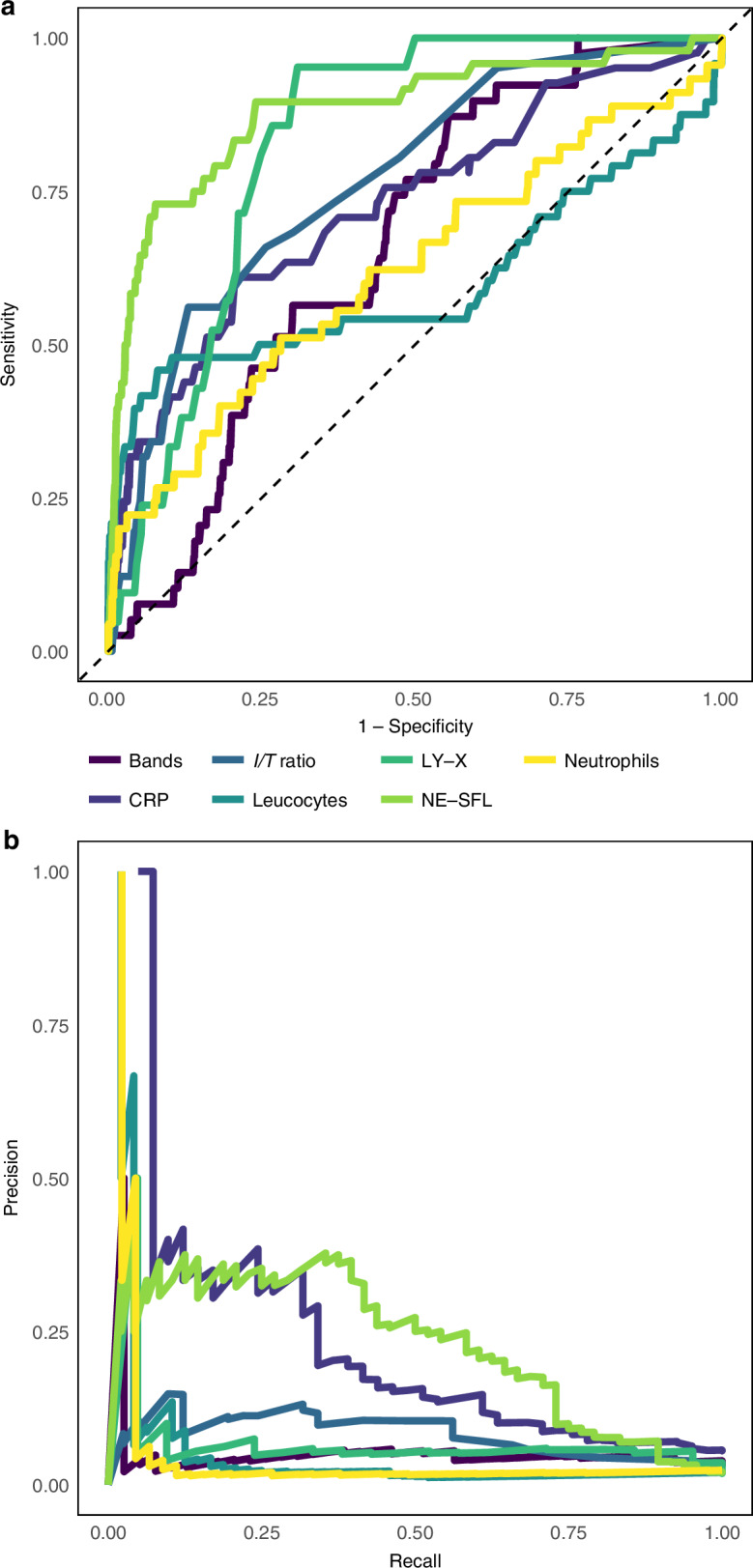
Receiver operating characteristic curve and precision-recall curve of NE-SFL, LY-X, classical hematological parameters and C-reactive protein. **a** Receiver operating characteristic curve, **b** Precision-recall curve. CRP C-reactive protein, I/T ratio immature-to-total ratio.

On the day of clinical suspicion, substantial differences emerged between sepsis/NEC cases and neonates in the reference group for most CPD parameters (Table 2). Similar findings were obtained in the population including neonates with comorbidities potentially affecting CPD (Supplementary Table S6). Neutrophil fluorescence intensity (NE-SFL) and lymphocytes cell complexity (LY-X) showed superior diagnostic accuracy to identify sepsis/NEC compared to other CPD parameters, leukocyte and neutrophil counts, bands, I/T ratio and CRP (Table 3, Fig. 3). In particular, NE-SFL had a sensitivity of 90%, a specificity of 76%, a positive predictive value (PPV) of 13%, a negative predictive value (NPV) of 99%, and an average precision (AP) of 0.20 (Table 3, Fig. 3). Including neonates with comorbidities potentially affecting CPD did not reduce diagnostic accuracy of CPD (Supplementary Table S7, Supplementary Fig. S2). We built three logistic regression models using the most performant CPD parameters, NE-SFL and LY-X, combined with leukopenia, and/or CRP (Supplementary Tables S8, S9, S10, and S11). All parameters showed a positive association with sepsis/NEC with a low multicollinearity among the predictors (Supplementary Tables S8 and S10). All models provided a slight improvement in diagnostic performance compared to NE-SFL alone, with no single model showing clear superiority over the others (Table 3, Supplementary Figs. S3 and S4, Tables S9 and S11).

**Table 2:** Cell population data in neonates from the reference group and patients with sepsis/necrotizing enterocolitis.

Parameter	Reference group	Sepsis/NEC group	*P* value
NE-SSC	149.9 (147.0–152.8)	148.7 (144.4–152.6)	0.08
NE-SFL	41.1 (38.8–44.0)	56.2 (46.5–63.7)	<0.001
NE-FSC	86.7 (84.2–89.1)	84.3 (80.9–86.2)	<0.001
NE-WX	354.0 (338.0–381.0)	358.0 (334.0–400.2)	0.61
NE-WY	789.0 (697.0–900.0)	904.5 (788.2–1123.5)	<0.001
NE-WZ	739.0 (710.0–770.8)	747.5 (718.2–794.0)	0.12
MO-X	116.2 (114.4–118.4)	118.7 (115.6–123.2)	<0.001
MO-Y	85.6 (80.1–91.4)	94.4 (85.7–103.8)	<0.001
MO-Z	69.1 (67.2–71.1)	69.8 (67.3–73.0)	0.30
MO-WX	278.0 (261.0–299.0)	302.5 (273.0–367.2)	<0.001
MO-WY	815.0 (740.2–903.0)	869.0 (710.0–997.2)	0.45
MO-WZ	679.5 (639.2–718.0)	683.5 (621.2–731.0)	0.88
LY-X^a^	78.3 (76.6–80.4)	81.3 (80.6–82.2)	<0.001
LY-Y^a^	58.8 (54.1–63.3)	65.4 (58.2–70.6)	<0.05
LY-Z^a^	58.7 (56.8–60.1)	59.7 (58.2–62.0)	<0.05
LY-WX^a^	500.0 (470.0–536.0)	492.0 (458.0–523.0)	0.30
LY-WY^a^	1000.0 (933.0–1143.8)	1225.0 (1078.0–1342.0)	<0.001
LY-WZ^a^	701.0 (664.0–804.0)	790.0 (690.0–864.0)	<0.05

Data are expressed as median (interquartile range). Cell Population Data are reported in arbitrary units of light scattering (ch).

^a^Data on 433/905 (48%) patients is provided, as data was not stored in 472 patients.

**Table 3:** Diagnostic accuracy of cell population data, classical hematological parameters, C-reactive protein, and combined models.

Parameter	AUROC	Cut off value	Sp%	Se%	PPV%	NPV%	AP
NE-SSC	0.57	147.2	73	46	7	97	0.01
NE-SFL	0.88	44.2	76	90	13	99	0.20
NE-FSC	0.70	85.7	63	73	8	98	0.01
NE-WX	0.52	422.5	93	21	10	97	0.03
NE-WY	0.68	888.5	73	58	8	98	0.05
NE-WZ	0.57	773.5	77	44	7	97	0.03
MO-X	0.67	117.2	64	71	8	98	0.16
MO-Y	0.71	93.6	81	56	11	98	0.09
MO-Z	0.54	72.8	90	27	11	97	0.06
MO-WX	0.69	322.5	89	46	14	98	0.05
MO-WY	0.53	962.5	86	35	9	97	0.03
MO-WZ	0.49	698.5	64	48	5	97	0.02
LY-X	0.83	79.8	69	95	11	100	0.06
LY-Y	0.66	64.8	80	57	11	98	0.04
LY-Z	0.63	61.7	92	38	16	97	0.05
LY-WX	0.57	525.5	31	86	5	98	0.04
LY-WY	0.75	1077.5	66	76	9	99	0.01
LY-WZ	0.65	753.5	66	62	7	98	0.04
Leukocytes (G/L)	0.60	6.3	92	46	19	98	0.04
Neutrophils (G/L)	0.62	3.6	72	51	7	97	0.06
Bands (G/L)	0.66	0.2	45	87	6	99	0.05
Leucopenia (G/L)	0.60	<5	97	33	31	97	0.04
Neutropenia (G/L)	0.62	<1.5	95	22	14	97	0.06
I/T ratio (%)	0.77	20	96	20	16	97	0.08
CRP (mg/L)	0.73	24.5	79	61	11	98	0.23
NE-SFL + LY-X + CRP (mg/L)	0.89	0.03	92	75	29	99	0.26
NE-SFL + LY-X + leucopenia (G/L)	0.89	0.02	90	77	25	99	0.27
NE-SFL + LY-X + leucopenia (G/L) + CRP (mg/L)	0.90	0.02	91	77	25	99	0.28

Cell population data are reported in arbitrary units of light scattering (ch).

*AUROC* area under the Receiver Operating Characteristic curve, *Se* sensitivity, *Sp* specificity, *PPV* positive predictive value, *NPV* negative predictive value, *AP* Average Precision, *CRP* C-reactive protein, *I/T ratio* immature-to-total ratio.

## Discussion

In this retrospective observational study, we established reference intervals for neutrophil, monocyte, and lymphocyte CPD in newborns, defining ranges from birth to postnatal day 28 across different gestational age groups. In an exploratory analysis of sepsis and NEC cases, we identified early alterations of most CPD parameters, suggesting that CPD could provide attractive biomarker candidates for the diagnosis of sepsis and NEC.

Previous studies have shown that hematological parameters vary with gestational and postnatal age, highlighting the importance of establishing reference intervals in newborns.^23–26,31^ Our study contributes to this body of knowledge by establishing reference intervals for CPD in a large cohort of hospitalized newborns. While reference intervals for CPD have been established in the adult population, to our knowledge, this is the first report on neonatal intervals.^32–34^ Furthermore, our findings highlight the considerable impact of gestational and postnatal age on neutrophil, monocyte, and lymphocyte CPD, revealing variations in complexity, fluorescence, and size across gestational age and throughout the neonatal period, contributing to increase our understanding of the developmental biology of blood leukocytes.

Reference intervals for CPD parameters, which capture the evolution of leukocyte subpopulation morphology and functionality, reveal trends that differ from those of absolute cellular counts.^24–26^ Across the three CPD subpopulations assessed in our study, we observed a reduction in the distribution width of most CPD parameters with increasing gestational age, probably indicating enhanced cellular homogeneity in more mature infants. In line with those findings, CPD distribution width reference intervals in adults are lower than those of full-term neonates, suggesting a developmental process towards greater cellular homogeneity.^32–34^ In contrast, each mean CPD parameter exhibited its own unique pattern of change with both gestational and postnatal age.

NE-SSC, an indicator of neutrophil cell complexity and granule content, progressively increases with gestational but not postnatal age, indicating that the maturation process of neutrophils could depend mainly on gestational age at birth.^35^ The reference intervals in full-term newborns are close to those observed in adults.^32–34^ NE-SFL, which depends on the RNA/DNA neutrophil content ratio, may reflect cellular immaturity or activation.^36,37^ In our results, NE-SFL shows complex changes across gestational and postnatal age, with reference intervals in full term newborns closely aligning with adult ranges, likely reflecting the dynamic interplay between neutrophil maturation and immune system activation in early life.^32–35,38^ NE-FSC, proportional to neutrophil size, also shows a complex pattern of changes with increasing gestational and postnatal age. As NE-FSC values are higher in adults, it could indicate that the maturation process in terms of neutrophil size extends beyond the neonatal period.^32–34^

MO-X, reflecting monocyte cellular complexity, exhibits complex variations according to gestational age at birth. Values in term newborns approach those observed in adults.^32–34^ MO-Y, which depends on the RNA and DNA content of monocytes, exhibits a complex pattern in relation to gestational age at birth. Neonatal values remain lower than those observed in the adult population, likely reflecting a reduced transcriptional ability in neonates, particularly in preterm infants.^32–34,39^ MO-Z, reflecting monocyte cellular size, remains relatively stable across different gestational and postnatal ages, aligning closely with the reference ranges established in adults.^32–34^

LY-X, indicating lymphocytic complexity and granularity, decreases with increasing gestational age at birth, while it remains relatively stable with increasing postnatal age. Adults present even lower reference ranges.^32–34^ LY-Y, which depends on RNA and DNA content, increases with gestational age at birth and subsequently exhibits a complex evolution during the neonatal period. Term newborns exhibit values comparable to adult ranges.^32–34^ This variation could be related to phenotypical changes, such as increased expression of transcription factors in more mature infants at birth and the rise in cytotoxic T/NK lymphocytes and B lymphocytes during the first postnatal weeks.^40,41^ LY-Z, reflecting the size of lymphocytes, remains stable across gestational ages at birth and throughout the neonatal period, with term neonates showing values lower than those observed in adults.^32–34^

In our exploratory analysis on sepsis and NEC, most CPD parameters show substantial differences between cases and neonates in the reference group at the onset of disease. NE-SFL and LY-X exhibit the highest diagnostic accuracy, which is superior to classical hematological parameters and CRP. NE-SFL has the strongest diagnostic potential for sepsis/NEC. The higher NE-SFL might be related to increased transcriptional activity in immature or activated neutrophils mobilized into peripheral blood during sepsis.^36,37^ Studies conducted in adults have identified a potential of NE-SFL to detect sepsis at the onset of symptoms, and a correlation with bacterial load.^36,42–45^ A pilot study showed higher NE-SFL in critically ill children with sepsis compared to those without infection.^46^ Few studies have investigated LY-X in sepsis, with conflicting results in adults.^42–44^ Our exploratory analysis identifies high LY-X, in newborns with sepsis/NEC, suggesting a potential role as a biomarker.

In line with previous studies, leukopenia, neutropenia, and elevated I/T ratio have low to moderate accuracy in detecting sepsis and NEC, with high specificity but low sensitivity and PPV.^27–29,47^ Similarly, CRP has moderate sensitivity and specificity, consistent with published findings, underscoring its limited utility as a standalone diagnostic marker.^48,49^ In our exploratory analysis, NE-SFL and LY-X provide a better trade-off between sensitivity and specificity, further supporting the potential of CPD parameters in the diagnosis of neonatal sepsis and NEC. Moreover, CPD offer several benefits compared to classical biomarkers. Since they are automatically generated with the CBC, they are available 24/7 without additional costs or extra sampling, a crucial feature for preterm newborns. In addition, as numerical data, CPD provide an objective, accurate, and faster alternative to manual differential counts.^18^

In our study, we evaluated three multivariate logistic regression models integrating the most accurate biomarkers from our univariate analysis, namely NE-SFL, LY-X, leukopenia, and CRP. While measuring multiple biomarkers concurrently has shown promise in diagnosing sepsis in adults, evidence supporting its use in neonatal sepsis remains limited.^43,50–54^ In our exploratory analysis, these models slightly improve specificity and PPV compared to NE-SFL alone, but the overall performance remains mostly unchanged, with PPV still limited.

This study benefits from its large population of hospitalized newborns, ensuring comprehensive and representative data. Reference intervals were rigorously defined following recommendations from neonatal literature.^23^ The use of mixed-effects models allowed to analyze the effects of gestational and postnatal age, while accounting for interindividual variability. This study has limitations. Although our large cohort provided an effective basis for determining reference intervals for CPD parameters, the small number of sepsis/NEC cases restricted our ability to evaluate their diagnostic performance, allowing only exploratory analyses in the early identification of sepsis/NEC. The monocentric and retrospective design limits the generalizability of the findings. As 30% of patients were excluded due to missing CPD parameters, this could have introduced some bias in our analyses. Due to the lack of prenatal data in our cohort, we could not exclude infants born from mothers with conditions known to influence neonatal leukocyte parameters. To minimize the impact of these potential confounders, we excluded neonates with any documented neutropenia.

## Conclusion

This study defines reference intervals for CPD in a large cohort of hospitalized newborns, enabling the interpretation of these preclinical parameters. We uncover unique trends in CPD based on gestational and postnatal age, providing a comprehensive assessment of the morphological characteristics of neutrophils, lymphocytes, and monocytes during the neonatal period. Our exploratory analysis suggests that CPD parameters hold potential for identifying patients who might require antibiotic treatment. Among CPD, NE-SFL emerges as a promising tool for decision-making in cases of sepsis or NEC.

## Supplementary information

SupplementaryData CPD revision

Supplementary Table S4

Supplementary Table S5

## Data Availability

The data that support the findings of this study are not openly available due to reasons of sensitivity and are available from the corresponding author upon reasonable request.
