# Supplementary material for: Neonatal leucocyte cell population data: reference intervals and relevance for detecting sepsis and necrotizing enterocolitis
Source: Pediatr Res. 2025 Jun 6;99(2):612–22. doi: 10.1038/s41390-025-04159-x (PMC12956581; doi:10.1038/s41390-025-04159-x)
Supplement: Supplementary file 1 — SupplementaryData CPD revision [file 41390_2025_4159_MOESM1_ESM.pdf]

Supplementary Material

Supplementary Table S1. Description of leukocyte Cell Population Data

CPD parameter	Description	Unit
NE-SSC	Neutrophil cell complexity	ch
NE-SFL	Neutrophil fluorescence intensity	ch
NE-FSC	Neutrophil cell size	ch
NE-WX	Distribution width of neutrophil cell complexity	
NE-WY	Distribution width of neutrophil fluorescence intensity	
NE-WZ	Distribution width of neutrophil cell size	
MO-X	Monocyte cells complexity	ch
MO-Y	Monocyte fluorescence intensity	ch
MO-Z	Monocyte cell size	ch
MO-WX	Distribution width of monocyte cells complexity	
MO-WY	Distribution width of monocyte fluorescence intensity	
MO-WZ	Distribution width of monocyte cell size	
LY-X	Lymphocyte cells complexity	ch
LY-Y	Lymphocyte fluorescence intensity	ch
LY-Z	Lymphocyte cell size	ch
LY-WX	Distribution width of lymphocyte cells complexity	
LY-WY	Distribution width of lymphocyte fluorescence intensity	
LY-WZ	Distribution width of lymphocyte cell size	

ch, arbitrary units of light scattering.

Supplementary Figure S1. Study Flow chart.

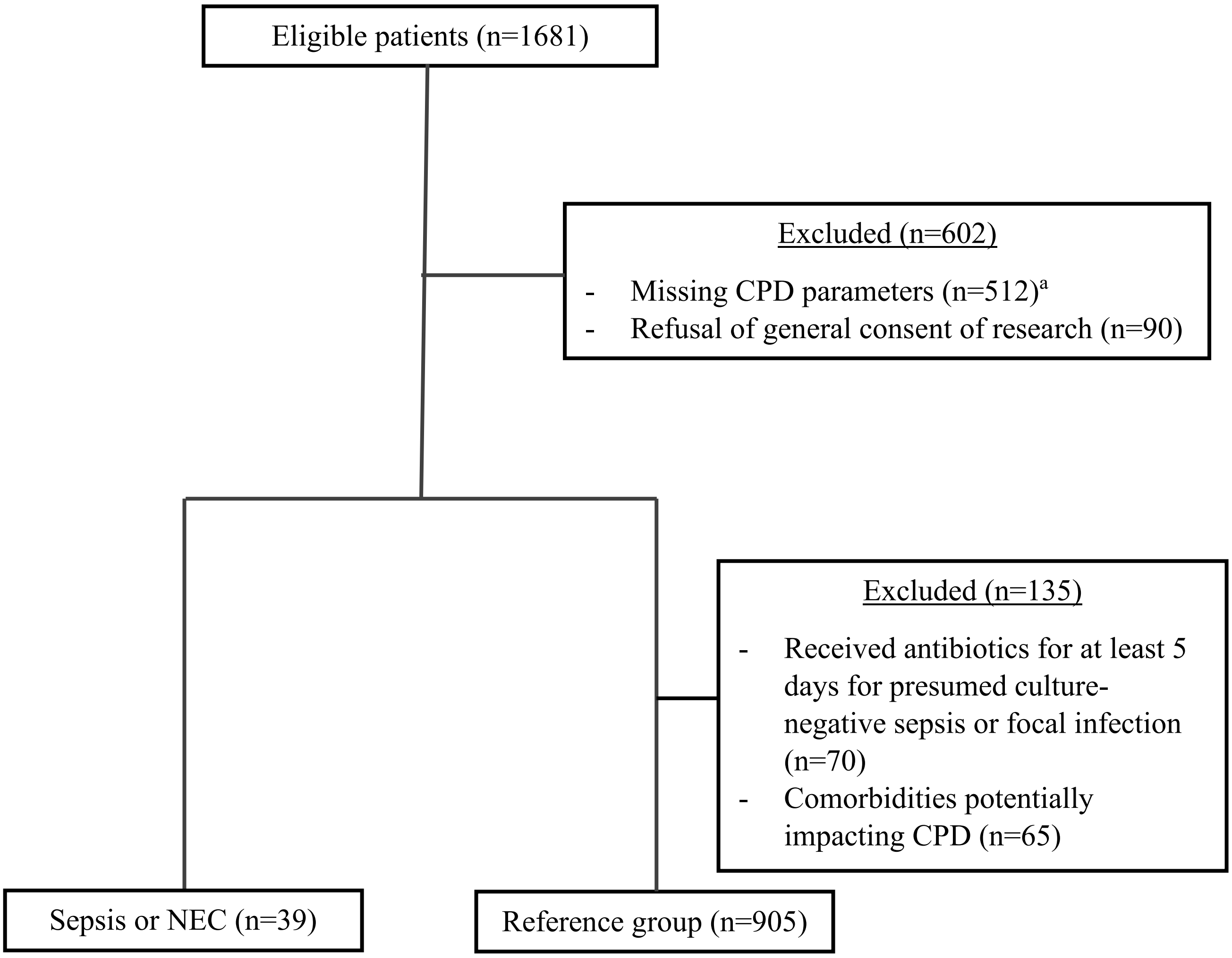

a. Missing CPD occurred as the hematology analyzer did not store any data during a specific time period (January 2022–May 2022).

Supplementary Table S2. Interval between suspicion time and Cell Population Data analysis

Patient number	CPD to BC interval (hours)	CPD to abdominal X-ray interval (hours)
1	0	-
2	-3	-
3	0	-
4[a]	0	8
5 [a]	-9	-11
6	10	-
7	0	-
8 [a]	-2	-2
9	58	-
10 [a]	5	5
11	55	-
12	0	-
13	44	-
14	84	-
15 [a]	-17	-17
16	0	-
17	0	-
18	15	-
19 [a]	0	0
20	10	-
21	2	-
22	1	-
23	0	-
24	-6	-
25	32	-
26	0	-
27	-11	-
28	-4	-
29	-11	-
30	0	-
31 [a]	-6	-10
32	0	-
33 [a]	0	0
34	-13	-
35 [a]	3	3
36	-17	-
37	0	-
38 [a]	31	31
39	0	-

CPD, Cell Population Data. BC, blood culture. Time elapsed between the blood culture and/or abdominal X-ray and the blood sample collection for CPD parameters. a. Necrotizing enterocolitis cases.

Supplementary Table S3. Demographic, clinical, and microbiological characteristics of patients, in a population including neonates with comorbidities potentially impacting Cell Population Data.

	Reference group n = 970	Sepsis/NEC group n = 39	P value
Postnatal age (days) at sepsis or NEC onset	—	9 (6-20)	—
Birth weight (kg)	2.8 (1.9-3.4)	0.9 (0.7-2.0)	<0.001
Gestational age (weeks)	37 (34-40)	28 (26-34)	<0.001
Female	414 (44)	23 (59)	0.06
Pathogens in blood culture proven sepsis	—	34 (87)	—
Escherichia coli	—	8 (24)	—
Coagulase-negative staphylococci	—	6 (18)	—
Staphylococcus aureus	—	4 (12)	—
Streptococcus agalactiae	—	3 (9)	—
Klebsiella pneumoniae	—	3 (9)	—
Klebsiella oxytoca	—	2 (6)	—
Candida albicans	—	2 (6)	—
Others pathogens[a]	—	6 (17)	—
Early-onset sepsis	—	8 (24)	—
Late-onset sepsis	—	26 (76)	—
Necrotizing enterocolitis	—	10 (26)	—

Continuous variables are reported as median (interquartile range). Categorical variables are reported as number (percent). Early-onset sepsis: onset before 72 hours of life; late-onset sepsis: onset after 72 hours of life.

a. *Achromobacter ssp., Bifidobacterium breve, Clostridium neonatale, Enterobacter cloacae, Enterococcus faecalis, Lacticaseibacillus rhamnosus.*

Supplementary Table S6. Cell Population Data in patients with sepsis/necrotizing enterocolitis and in a population including neonates with comorbidities potentially impacting Cell Population Data.

Parameter	Reference group	Sepsis/NEC group	P value
NE-SSC	149.8 (146.9-152.8)	148.7 (144.4-152.6)	0.10
NE-SFL	41.3 (39.0-44.1)	56.2 (46.5-63.7)	<0.001
NE-FSC	86.7 (84.2-89.1)	84.3 (80.9-86.2)	<0.001
NE-WX	355.0 (338.0-381.0)	358.0 (334.0-400.2)	0.67
NE-WY	792.0 (699.0-906.0)	904.5 (788.2-1123.5)	<0.001
NE-WZ	739.0 (709.0-771.0)	747.5 (718.2-794.0)	0.09
MO-X	116.2 (114.4-118.4)	118.7 (115.6-123.2)	<0.001
MO-Y	85.9 (80.3-92)	94.4 (85.7-103.8)	<0.001
MO-Z	69.2 (67.2-71.2)	69.8 (67.3-73.0)	0.35
MO-WX	279.0 (261.0-301.0)	302.5 (273.0-367.2)	<0.001
MO-WY	820.0 (741.0-909.0)	869.0 (710.0-997.2)	0.54
MO-WZ	678.0 (638.0-717.0)	683.5 (621.2-731.0)	0.95
LY-X[a]	78.4 (76.6-80.5)	81.3 (80.6-82.2)	<0.001
LY-Y[a]	58.8 (54.0-63.5)	65.4 (58.2-70.6)	0.06
LY-Z[a]	58.6 (56.8-60.1)	59.7 (58.2-62)	<0.05
LY-WX[a]	502.0 (471.0-539.8)	492.0 (458.0-523.0)	0.22
LY-WY[a]	1009.0 (937.0-1160.0)	1225.0 (1078.0-1342.0)	<0.001
LY-WZ[a]	705.0 (665.0-809.0)	790.0 (690.0-864.0)	<0.05

Data are expressed as median (interquartile range). Cell Population Data are reported in arbitrary units of light scattering (ch).
a. Data on 467/970 (48%) patients is provided, as data was not stored in 503 patients.

Supplementary Table S7. Diagnostic accuracy of Cell Population Data compared to classical hematological parameters and C-reactive protein, in a population including neonates with comorbidities potentially impacting Cell Population Data.

Parameter	AUROC	Cut off value	Sp%	Se%	PPV %	NPV%	AP
NE-SSC	0.57	147.2	72	46	6	97	0.01
NE-SFL	0.88	49.8	92	73	28	99	0.20
NE-FSC	0.70	85.7	62	73	7	98	0.01
NE-WX	0.52	422.5	92	21	10	97	0.03
NE-WY	0.67	888.5	72	58	8	98	0.05
NE-WZ	0.57	773.5	77	44	7	97	0.02
MO-X	0.67	117.2	64	71	7	98	0.16
MO-Y	0.70	93.6	80	56	10	98	0.09
MO-Z	0.54	72.8	89	27	10	97	0.06
MO-WX	0.68	322.5	87	46	12	97	0.05
MO-WY	0.53	962.5	85	35	9	97	0.03
MO-WZ	0.50	698.5	64	48	5	97	0.02
LY-X	0.68	79.8	85	42	10	97	0.06
LY-Y	0.65	64.8	80	57	10	98	0.04
LY-Z	0.63	61.7	92	38	16	97	0.04
LY-WX	0.58	525.5	33	86	5	98	0.01
LY-WY	0.73	1,181.5	78	62	10	98	0.05
LY-WZ	0.64	753.5	64	62	7	98	0.04
Leukocytes (G/L)	0.60	6.3	92	46	18	98	0.04
Neutrophils (G/L)	0.62	3.6	72	51	7	97	0.06
Bands (G/L)	0.66	0.2	44	87	6	99	0.05
Leucopenia (G/L)	0.60	<5	97	33	29	97	0.04
Neutropenia (G/L)	0.62	<1.5	94	22	14	97	0.06
I/T ratio (%)	0.76	20	96	20	16	97	0.08
CRP (mg/L)	0.71	8.9	87	65	0.17	98	0.03
NE-SFL + LY-X + CRP	0.89	0.03	94	73	33	99	0.24
NE-SFL + LY-X + Leucopenia	0.89	0.02	90	77	25	99	0.22
NE-SFL + LY-X + CRP + Leucopenia	0.89	0.03	93	75	32	99	0.23

Cell Population Data are reported in arbitrary units of light scattering, ch; AUROC, area under the Receiver Operating Characteristic curve; Se, sensitivity, Sp, specificity; PPV, positive predictive value; NPV, negative predictive value; AP, Average Precision; CRP, C-reactive protein; I/T ratio, immature-to-total ratio.

Supplementary Figure S2. Receiver Operating Characteristic curve and Precision-Recall Curve of NE-SFL, LY-X, classical hematological parameters and C-reactive protein, in a population including neonates with comorbidities potentially impacting Cell Population Data.

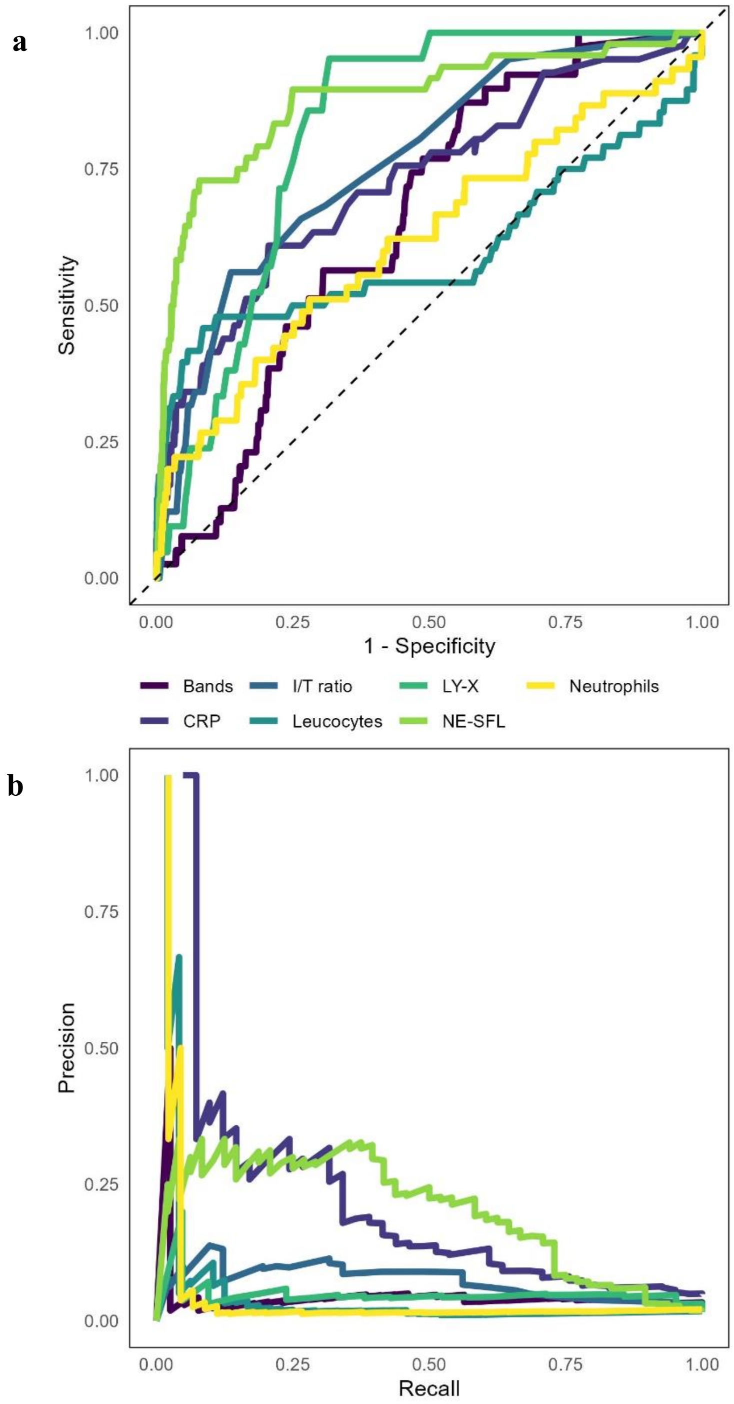

a) Receiver Operating Characteristic curve; b) Precision-Recall Curve
CRP, C-reactive protein; I/T ratio, immature-to-total ratio.

Supplementary Table S8. Regression coefficient results and Variance Inflation Factor for combined models, in the study population.

Model	Variable	Estimate	Std. Error	P value	VIF
NE-SFL + LY-X + CRP	Intercept	-25.85	5.04	<0.001	
	NE-SFL	0.14	0.02	<0.001	1.32
	LY-X	0.19	0.06	<0.01	1.00
	CRP	0.01	0.004	<0.05	1.33
NE-SFL + LY-X + Leucopenia	Intercept	-24.84	4.78	<0.001	
	NE-SFL	0.14	0.02	<0.001	1.09
	LY-X	0.18	0.06	<0.01	1.00
	Leucopenia	1.46	0.41	<0.001	1.09
NE-SFL + LY-X + CRP + Leucopenia	Intercept	-23.41	4.23	<0.001	
	NE-SFL	0.13	0.02	<0.001	1.38
	LY-X	0.18	0.06	<0.01	1.01
	CRP	0.01	0.005	0.11	1.41
	Leucopenia	1.05	0.43	<0.01	1.16

VIF, Variance Inflation Factor; CRP, C-reactive protein.

Supplementary Table S9. Cross-validation results for combined models in the study population.

Model cross validation	Mean AUROC	Mean Se%	Mean Sp%
NE-SFL + LY-X + CRP	0.90	17	99
NE-SFL + LY-X + Leucopenia	0.90	13	99
NE-SFL + LY-X + CRP + Leucopenia	0.90	15	99

AUROC, area under the Receiver Operating Characteristic curve; Se, sensitivity, Sp, specificity; CRP, C-reactive protein.

Supplementary Figure S3. Receiver Operating Characteristic curve and Precision-Recall Curve of three logistic regression model combining NE-SFL, LY-X, C-reactive protein, and leukopenia in the study population.

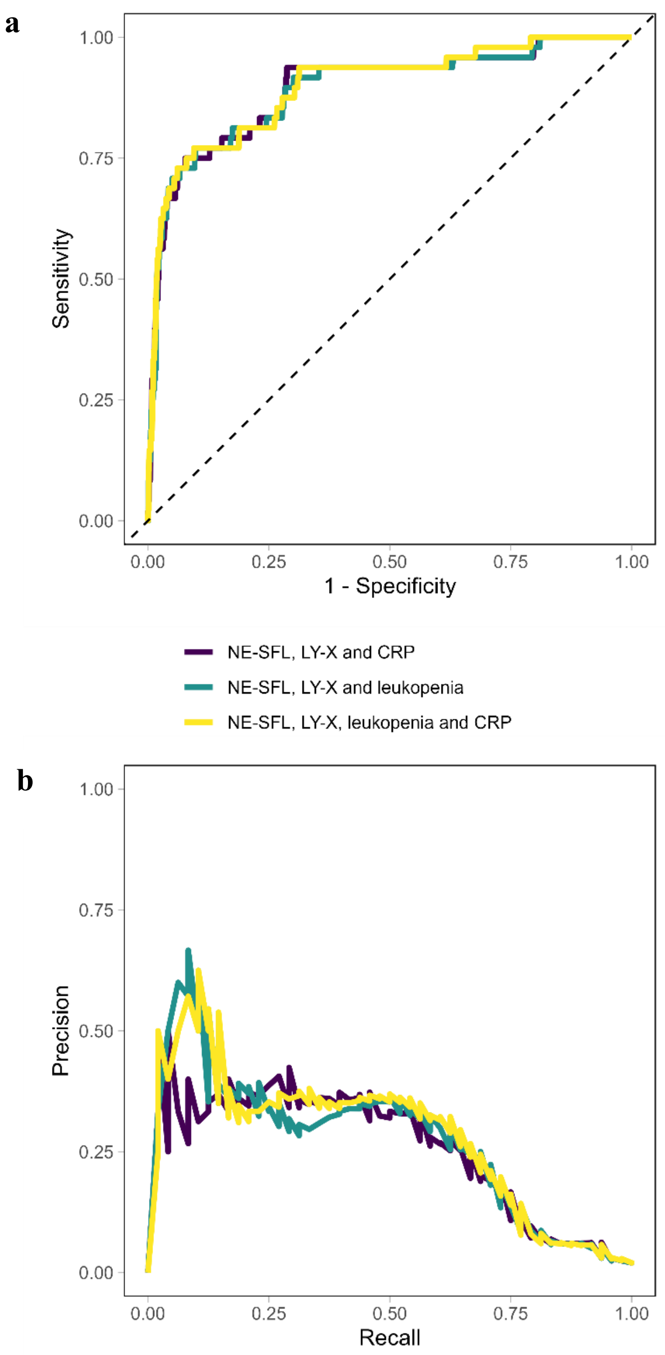

a) Receiver Operating Characteristic curve; b) Precision-Recall Curve
CRP, C-reactive protein; I/T ratio, immature-to-total ratio.

Supplementary Table S10. Regression coefficient results and Variance Inflation Factor for combined models, in a population including neonates with comorbidities potentially impacting Cell Population Data.

Model	Variable	Estimate	Std. Error	P value	VIF
NE-SFL + LY-X + CRP	Intercept	-23.81	4.23	<0.001	
	NE-SFL	0.13	0.02	<0.001	1.27
	LY-X	0.17	0.05	<0.01	1.01
	CRP	0.01	0.00	<0.01	1.28
NE-SFL + LY-X + Leucopenia	Intercept	-23.53	4.15	<0.001	
	NE-SFL	0.14	0.02	<0.001	1.15
	LY-X	0.16	0.05	<0.01	1.00
	Leucopenia	1.23	0.41	<0.01	1.16
NE-SFL + LY-X + CRP + Leucopenia	Intercept	-23.41	4.23	<0.001	
	NE-SFL	0.12	0.02	<0.001	1.39
	LY-X	0.17	0.05	<0.01	1.01
	CRP	0.01	0.00	<0.01	1.31
	Leucopenia	1.05	0.43	<0.05	1.19

VIF, Variance Inflation Factor; CRP, C-reactive protein

Supplementary Table S11. Cross-validation results for combined models, in a population including neonates with comorbidities potentially impacting Cell Population Data.

Model cross validation	Mean AUC	Mean Se%	Mean Sp%
NE-SFL + LY-X + CRP	0.89	100	11
NE-SFL + LY-X + Leucopenia	0.90	100	13
NE-SFL + LY-X + CRP + Leucopenia	0.89	100	15

AUROC, area under the Receiver Operating Characteristic curve; Se, sensitivity, Sp, specificity; CRP, C-reactive protein.

Supplementary Figure S4. Receiver Operating Characteristic curve and Precision-Recall Curve of three logistic regression model combining NE-SFL, LY-X, C-reactive protein, and leukopenia, in a population including neonates with comorbidities potentially impacting Cell Population Data.

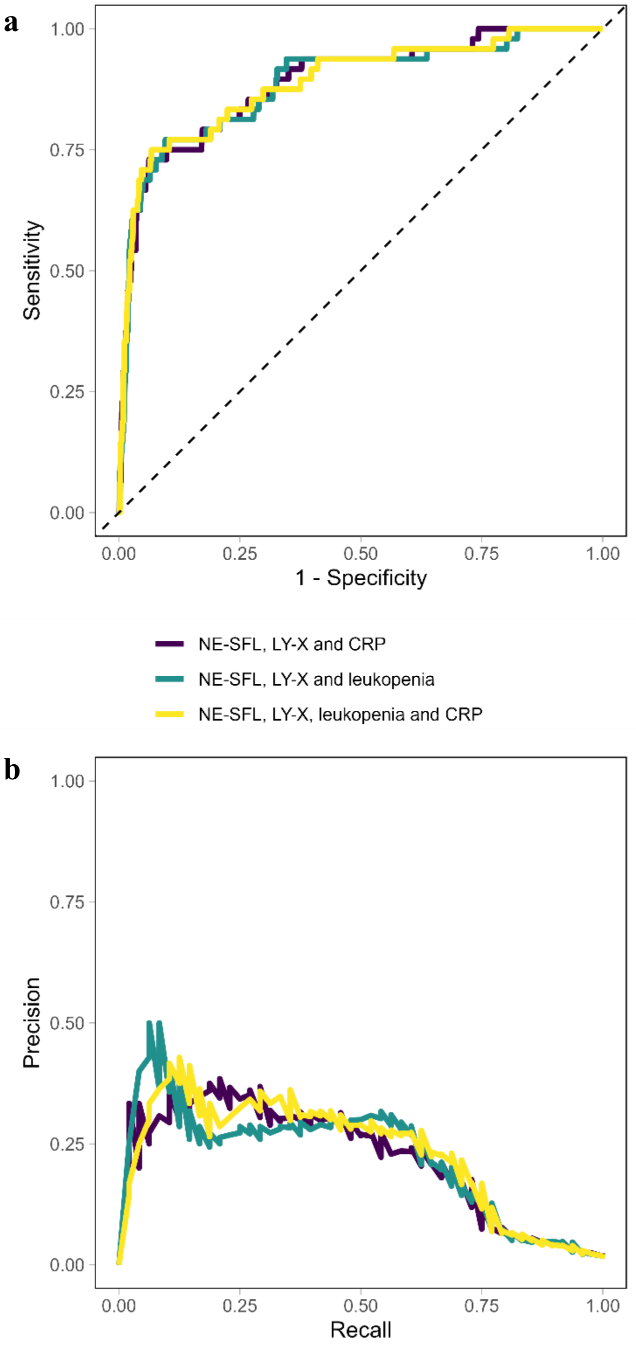

a) Receiver Operating Characteristic curve; b) Precision-Recall Curve
CRP, C-reactive protein.
